# Serological, Molecular and Culture-Based Diagnosis of Lentiviral Infections in Small Ruminants

**DOI:** 10.3390/v13091711

**Published:** 2021-08-27

**Authors:** Aphrodite I. Kalogianni, Ioannis Stavropoulos, Serafeim C. Chaintoutis, Ioannis Bossis, Athanasios I. Gelasakis

**Affiliations:** 1 Laboratory of Anatomy and Physiology of Farm Animals, Department of Animal Science, School of Animal Biosciences, Agricultural University of Athens (AUA), Iera Odos 75 Str., 11855 Athens, Greece; 2 Laboratory of Animal Husbandry, Department of Agricultural Sciences, School of Agriculture, Forestry and Natural Resources, Aristotle University of Thessaloniki (AUTh), 54124 Thessaloniki, Greece; istavrop@agro.auth.gr (Ι.S.); bossisi@agro.auth.gr (I.B.); 3 Diagnostic Laboratory, School of Veterinary Medicine, Faculty of Health Sciences, Aristotle University of Thessaloniki (AUTh), 11 Stavrou Voutyra Str., 54627 Thessaloniki, Greece; schainto@vet.auth.gr

**Keywords:** small ruminant lentiviruses, maedi-visna, caprine arthritis-encephalitis, diagnosis, serological methods, molecular methods, ELISA, PCR, cell cultures

## Abstract

Small ruminant lentiviruses (SRLVs) infections lead to chronic diseases and remarkable economic losses undermining health and welfare of animals and the sustainability of farms. Early and definite diagnosis of SRLVs infections is the cornerstone for any control and eradication efforts; however, a “gold standard” test and/or diagnostic protocols with extensive applicability have yet to be developed. The main challenges preventing the development of a universally accepted diagnostic tool with sufficient sensitivity, specificity, and accuracy to be integrated in SRLVs control programs are the genetic variability of SRLVs associated with mutations, recombination, and cross-species transmission and the peculiarities of small ruminants’ humoral immune response regarding late seroconversion, as well as intermittent and epitope-specific antibody production. The objectives of this review paper were to summarize the available serological and molecular assays for the diagnosis of SRLVs, to highlight their diagnostic performance emphasizing on advantages and drawbacks of their application, and to discuss current and future perspectives, challenges, limitations and impacts regarding the development of reliable and efficient tools for the diagnosis of SRLVs infections.

## 1. Introduction

Small ruminant lentiviruses (SRLVs) are a group of non-oncogenic viruses of the family *Retroviridae*, that infect both sheep and goats causing chronic, incurable, inflammatory diseases known as maedi-visna (MV) and caprine arthritis-encephalitis (CAE) [1]. SRLVs are characterized by high genetic variability among genotypes (genotypes A (subtypes A1–A22), B (subtypes B1–B5), C, and E (subtypes E1–E2)) [2,3,4]. Nevertheless, they display similar pathogenesis affecting lungs, mammary gland, central nervous system and joints, and similar tropism by infecting monocytes/macrophages and dendritic cells [2,3,5]. Clinical manifestations of the disease in chronically infected animals include pneumonia and mastitis, encephalitis and arthritis; however, most infected animals are usually asymptomatic due to the slow and progressive evolution of the infection [1,5,6,7]. The primary source of infection for newborn lambs is the consumption of colostrum and milk from infected ewes (lactogenic route) [8,9]. Horizontal transmission via respiratory secretions is also significant, especially in intensively reared small ruminants [10,11], whereas transplacental transmission [12] and transmission via semen during mating or artificial insemination are also possible, but their significance and extent has not been thoroughly studied [8,13].

The economic impact of the SRLVs global spreading on the small ruminant sector has not yet been fully elucidated; however, it is widely recognized as a major cause of (i) increased replacement rate, resulting from the involuntary culling of animals with clinical disease, (ii) decreased lambs’ growth rate and milk production (quantitatively and qualitatively) due to the adverse effects on the secretory capacity of the mammary gland and (iii) restrictions in breeding stocks and semen trading [2,14,15,16].

Considering the lack of efficient treatment or vaccination, early and accurate diagnosis of SRLVs infections is paramount for the successful implementation of control programs, the eradication of MV and CAE, and the accreditation of SRLV-free regions and farms. Diagnosis of SRLVs is based either on the detection of SRLV-specific antibodies with serological tests such as agar gel immunodiffusion (AGID), enzyme-linked immunosorbent assay (ELISA), radioimmunoprecipitation (RIPA), radioimmunoassay (RIA) and Western blot (WB), or on the detection of viral genome with molecular assays (e.g., polymerase chain reaction (PCR), real time PCR (qPCR)) and virus isolation in cell cultures [17]. Viral capsid and matrix proteins (p25CA, p28CA, p14NC and p16MA), and envelope glycoproteins (gp135SU, gp46TM) coded by the *gag* and *env* genes, respectively, are commonly used as antigens for the detection of SRLV-specific antibodies, whereas long terminal repeats (LTRs) of proviral DNA, and conserved regions in the *pol, gag* and *env* genes are used as targets for primers used in molecular assays [6,18,19]. 

Lack of a “gold standard” assay for the early diagnosis of SRLVs infections, has led to various types and combinations of serological and molecular assays being utilized in eradication programs around the world with variable efficacy [20,21,22,23,24,25,26,27,28,29,30]. The limited success of the currently applied programs to control the disease implies that some of the infected animals evade diagnosis acting as virus reservoirs for the establishment of re-infections. Τhis situation perpetuates the economic impact of SRLVs infections, increases the uncertainty and the cost of the invested resources for SRLVs eradication, and last but not least, reduces the willingness of farmers to participate in control programs. 

Currently, universally applicable diagnostic tools are not available, and the development of highly sensitive and specific diagnostic protocol is a priority. Development of efficient diagnostic tools is a challenging task due to (i) the genetic variability of SRLVs associated with mutations, recombination and cross-species transmission, and (ii) the peculiarities of small ruminants’ humoral immune response regarding late seroconversion, intermittent and epitope-specific antibody production. The objectives of this review paper were to summarize the available diagnostic assays and methods routinely used in SRLVs control programs emphasizing on their applications, advantages, and drawbacks, and to describe and discuss current and future perspectives, challenges, limitations and impacts regarding the development of reliable and efficient diagnostic tools for SRLVs.

## 2. Diagnosis of Small Ruminant Lentiviral Infection

### 2.1. Serological Methods

#### 2.1.1. Agar Gel Immunodiffusion (AGID)

AGID test had been previously recommended from the OIE as the method of choice for SRLVs routine screening for animal trading and eradication programs against MV and CAE [18]. However, after the validation and wide application of commercial ELISAs, AGID test has been used mainly as a confirmatory test rather than screening purposes [1,27]. More precisely, two AGID tests were used in voluntary national MV control program in Finland (AGID kit Institut Pourquier MV/CAEV for screening, and AGID Maeditect 1000, Central Veterinary Laboratory, UK for confirmation of positive samples) [23] and AGID kit Maeditect (Veterinary Laboratories Agency, Weybridge, UK) has been used initially as screening test and later as confirmatory test in ELISA positive samples (CAEV/MAEDI-VISNA kit, Institut Pourquier, Montpellier, France) in control program in Norway [31,32]. Similarly, in UK control programs, Maeditect and Capriclear AGID tests (Central Veterinary Laboratory, Weybridge, UK) have been used as initial diagnostic methods due to their high specificity, followed by indirect ELISA Elitest MVV/CAEV (Hyphen BioMed, Neuville-sur-Oise, France) as routine screening assays to improve sensitivity [22]. The most commonly used antigens in AGID tests are the MVV p25 or the CAEV p28 capsid antigen (CA) and the envelope glycoprotein gp135 (SU) obtained from cell culture supernatants infected with certain viral strains (e.g., CAEV-63 and MVV WLC1) [17,18,19]. The performance of AGID test depends mainly on viral strains and specific viral antigens used, as agar gel precipitation requires multiple binding sites between antibodies and viral epitopes [17,19]. In goats, AGID sensitivity ranges from 56.0 to 92.0% and the specificity is 100.0%, whereas the respective values in sheep range from 76.3 to 99.3% and from 98.3 to 99.4% depending on the viral antigens and the confirmatory methods utilized [17,18,19]. Although cross-reactivity has been reported [27], the partially conserved epitopes among MV and CAE viral strains and the different immune responses of sheep and goats regarding immunodominant epitopes hinders the robust interaction of antibodies with the selected epitopes [17,19]. In addition, the combination of viral antigens may lead to higher sensitivity, since the humoral immune response fluctuates depending on the infection stage; antibodies against gp135 are predominant in chronically infected animals, whereas antibodies against capsid antigens (p28/p25) are present during the early infection stages [17,27,33]. Although commercial AGID kits are available (e.g., Maeditect kit, APHA Scientific, Addlestone, Surrey, United Kingdom; AGID CAEV P28, IDEXX, Westbrook, ME, USA) [27], the use of local strains can further improve the diagnostic performance of the method; however, this approach is laborious and increases the cost [19], as it requires costly equipment and consumables for the propagation of the virus in cell cultures. Furthermore, AGID is time-consuming, as the results are usually read after a 24–48-h incubation, and specialized personnel is required to visually interpret precipitin lines formed in the agar gel. Moreover, AGID’s low sensitivity does not favor its widespread use as a routine screening method [1,18,19,34]. On the other hand, its high specificity enhances its use as a confirmatory test (especially for ELISAs).

#### 2.1.2. Enzyme-Linked Immunosorbent Assay (ELISA)

ELISAs have been widely exploited in SRLVs control programs for the screening of sheep and goat populations. For example, in MVV control program in Aragón of Spain sheep serum samples have been tested by ELISA Elitest MVV/CAEV (Hyphen BioMed, Neuville-sur-Oise, France) [24], whereas in Dutch national MVV control program a complex-trapping blocking (CTB) ELISA of specific epitopes on p28 capsid protein has been exploited [35]; in compulsory CAEV eradication program in South Tyrol of Italy, ELISA CAEV/MAEDI-VISNA kit of Institut Pourquier (Montpellier, France) has been used from 2007 until 2011, before its replacement by ELISA IDEXX MVV/CAEV p28 Ab Screening Test (IDEXX, France) [36], whereas ΕLISA kit CAEV/MVV Total Ab (Idexx Switzerland AG, Liebefeld-Bern, Switzerland), and a home-made surface subunit SU5 peptides ELISA have been used as a screening method and a predictor of lentiviruses subtypes, respectively, in Swiss CAEV eradication program [37,38,39,40]. Despite the fact that its performance is not universally constant, ELISA remains a user-friendly, low-cost, semi-quantitative diagnostic test, with sufficient repeatability and, in most cases, sensitivity and specificity [18,34]. Both the commercially available kits (see Table 1) and in-house assays belong either to the indirect or to the competitive assay type for the detection of circulating antibodies in infected animals. In the indirect ELISA assays, antigens can be the whole virus, recombinant proteins, or synthetic peptides, whereas in the competitive assays, combinations of monoclonal antibodies are utilized for competition with sera antibodies for the coated viral antigens. Although ELISA is the most commonly used diagnostic test, scarcity of efficient validation protocols using at least one reference standard method (RIPA, or WB), according to the guidelines of OIE [17], constitutes the major flaw in the process of being officially recognized as valid and reliable screening assays. Although many ELISAs have been tested and reported for SRLVs detection [18], only a few have been validated for their high sensitivity compared to reference methods [17,19]; namely, (i) an indirect whole virus (OLV 130/91 strain) ELISA and a recombinant transmembrane (r-TM) ELISA (strain K1514) compared to AGID test (OLV 130/91 strain) and WB in sheep samples [41], (ii) an indirect whole virus (Canadian CAEV strain) ELISA compared to AGID test, WB and fixed-cell fluorescent antibody test in goat samples [42], (iii) the competitive ELISA CAEV-63 SU (surface envelope SU of the 79-63 CAEV isolate) of VMRD inc. compared to RIPA in sheep and goat samples [43,44], and (iv) the competitive CTB ELISA of Dutch control program has been validated against CAEV-63 AGID and ZZV1050 AGID tests for goat and sheep samples, respectively [45]. Only one indirect ELISA with capsid (CA) and transmembrane (TM) peptides (Elitest-MVV, HYPHEN Biomed, Neuville-sur-Oise, France and Pourquier) has been validated compared to OPPV WLC1 AGID test in sheep samples, according to OIE criteria [19,46]. In any case, for the objective assessment of its sensitivity, validation of an ELISA test should be conducted against reference sera standards with viral antigens of similar or variable strains coated on the ELISA plates. A considerable advantage of ELISAs when compared to other serological methods is the capability to be applied in various biological samples such as blood serum and plasma, and milk [47,48,49,50,51,52,53]. Among these samples, milk seems to be the most ambiguous sample matrix given that several factors may adversely affect the reliable diagnosis, such as the progressive reduction of antibodies throughout the lactation, the occurrence of false positive background signals in cases of mastitis, colostrum, increased milk fat content or even the specific immune response of the mammary gland depending on the infection stage [47,52]. ELISAs fluctuate between high sensitivity and low specificity and vice versa; for example, high sensitivity of competitive ELISAs due to the use of undiluted sera is usually combined by low specificity [19,43]. In general, the unsatisfactory diagnostic performance of ELISAs are mainly attributed to: (i) the unfavorable combination of antigen used in the test with the infection stage, as the production of antibodies against matrix and capsid proteins (e.g., p25, p28, and p16) during early infection stages precedes the production of other antibodies; on the contrary they are almost eliminated at later stages in the infected animals, where antibodies against gp46 and gp135 prevail [27,54,55,56], (ii) the antigenic distance between the viral strain used in the development of the assay and the infecting strain of the examined animals; although SRLVs are characterized by cross-reactivity [57,58], homologous humoral immune response in strain-specific epitopes reduces dramatically the sensitivity of ELISA test and therefore, leads to misdiagnosis [37,54,59,60], (iii) the late seroconversion of animals, the fluctuation of antibody response during animal’s life and the alternations between viremia and humoral immune responses [15,18,52,61], and (iv) the animal species; in goats, for example, a more robust reactivity against transmembrane glycoproteins compared to capsid proteins has been observed [37,55]. Therefore, except for the impediments arising from virus nature and the immunopathological mechanisms, a critical endeavor for the enhancement of serological diagnosis performance is to enrich the antigenic design of ELISA and improve its negative predictive value. The use of whole virus, incorporation of multiple antigens and synthetic peptide combinations, and genotype-specific immunodominant epitopes have been proposed for the extension of the antigenic spectrum and the amplification of the detection capacity of the assay [54,56,57,58,60,62,63]. 

#### 2.1.3. Other Serological Methods

RIPA, RIA and WB are usually used as “gold standard” methods. RIPA and RIA rely on the conformation of antibody-epitope complexes like in the AGID method; however, in these assays, the antigens (RIPA) and the antibodies (RIA) are ^35^S-labelled, increasing their sensitivity [19,67]. WB uses viral antigens, usually whole virus, which are separated in reducing sodium dodecyl sulfate polyacrylamide electrophoresis gels (SDS PAGE gels), transferred to nitrocellulose membranes and subsequently incubated with animal sera that potentially contain antibodies that recognize and bind to the separated viral antigens [19,34]. The denaturing conditions of WB instead of the native conditions in RIPA and AGID, favor the detection of specific antibodies binding to linear epitopes of CA, MA and TM proteins [19,39]. Despite their high sensitivity and specificity, RIPA, RIA and WB are not suitable for use in large-scale surveillance programs, but they are rather exploited as reference tests, since they are costly and time-consuming assays applied in specialized diagnostic laboratories by trained staff [18,49]. However, a WB technique (MVV strain ZZV 1050) has been used in the national MV control programs in the Netherlands and in Switzerland as confirmatory method of ELISA positive samples [35,39]. Nevertheless, the use of RIPA, RIA and WB for the validation of new diagnostic tests or for the confirmation of ELISA results, should not be considered a priori infallible, as both false positive results (due to nonspecific cross-reactivity) or false negative (due to weak affinity of circulating antibodies for epitopes of viral antigens) have been reported [18].

### 2.2. Molecular Methods

#### 2.2.1. PCR

SRLV proviral DNA can be detected in samples of peripheral blood mononuclear cells, colostrum and milk, bronchoalveolar fluid and lungs, mammary gland, carpal synovial membranes, brain, and other secondary tissue targets such as bone marrow, spleen, lymph nodes, testicles, ovaries, uterus, heart, kidneys and liver [19,47,48,51,52,56,61,68,69,70,71,72,73,74]. The presence of SRLV genetic material has been, also, reported in air and water samples collected from sheep farms, highlighting the potential for horizontal transmission of SRLVs [75]. After the development of the first successful PCR protocol applied for the detection of CAEV and MVV [76], remarkable progress has been made resulting in more sophisticated and reliable molecular diagnostic protocols. Except for the conventional PCR, other PCR techniques have been developed to improve the sensitivity, specificity and accuracy of molecular diagnostics. Indeed, combination of PCRs for different genomic regions, multiplex PCRs, (semi-)nested PCRs, and real-time PCRs have been exploited with contradictory results. The diagnostic performances of some of the PCR techniques used for SRLVs detection are summarized in Table 2. Primer sequences can be found in detail in supplementary material (Table S1).

For the application of PCR, DNA extracted mainly from peripheral blood leucocytes (PBLs) or mononuclear cells (PBMCs) or milk cells is used, while DNA extracted from tissues is less frequently utilized for confirmatory purposes; the possibility of detecting viral RNA by applying reverse transcription PCR is null, as circulating cell-free virions are usually non-detectable; however, it could be used to study horizontal virus transmission [19,34,61]. On a routine basis, DNA (genomic or/and episomal) is extracted either by commercial kits or via in-house methods from PBLs or PBMCs as monocytes/macrophages and dendritic cells are the only cells known to support replication of SRLVs. Major determinants for the selection of a DNA-extraction protocol are the time required, yield and quality of the extracted DNA [87]. In addition, a commercial qPCR kit (EXOone Maedi Visna CAEV oneMix, Exopol, Spain) is available for the diagnosis of SRLVs genotypes A, B, and E exhibiting higher sensitivity from serological methods and home-made *gag* PCR in diagnosis in field samples [63]. The major advantage of PCR technologies compared to the serological methods is the early detection of the SRLVs infection, preceding the production of antibodies which may occur months or years later [2]. Nonetheless, low viral load of infected animals may hinder the detection of proviral DNA resulting in false negative results and reduced sensitivity [67]. Decreased viral load is indicative of low number of infected monocytes [5,88] or restricted viral replication due to humoral immune response which probably acts protective for the infected animals [5,52,61]. Moreover, the high mutation rate of SRLVs due to the low fidelity of the virion’s reverse transcriptase and the frequently observed recombinations [1,89] undermine the diagnostic performance of PCR. To achieve sufficient specificity, the primers have to be designed for conserved regions of the viral genome, avoiding the *env* gene which is less conserved among genotypes [18,90]. On the other hand, the problem of virus genetic variability can be mitigated by the use of degenerate primers expanding the detection range and improving the sensitivity of the method [80,84,91]. In infected animals, false negative results of PCR could be linked to co-existence of multiple SRLVs mutants in an infected population. Although the development of universally applicable PCR assays may be extremely difficult due to the aforementioned obstacles, evidence-based modification of the protocols for the detection of local strains could be a realistic target in the field of SRLVs diagnostics. This is a necessary step when planning SRLVs surveillance programs, demanding (i) genotyping, sequencing, and phylogenetic analyses of the relevant strains, (ii) designation of specific and widely applicable primers, and (iii) the development of sensitive and specific PCR protocols with the potential and the capacity to be applied in a specific geographical region (with available specialized laboratory infrastructures, equipment, and staff).

#### 2.2.2. Other Molecular Assays

Heteroduplex mobility assay (HMA) usually follows the PCR amplification for the classification of the detected strains in comparison to the reference strains and for the assessment of the homogeneity of strains detected in a region or a flock [79,92,93,94,95]. It is a qualitative technique and a valuable tool to study the molecular epidemiology of SRLVs. In addition, loop-mediated isothermal amplification (LAMP) and recombinase polymerase amplification lateral flow dipstick (RPA-LFD) techniques have been lately applied with success for CAEV diagnosis [96,97,98]. Although results seem promising when compared to “traditional” serological and molecular techniques, more studies are needed for the validation of the diagnostic performance of these innovative techniques in a wider spectrum of viral strains.

### 2.3. Cell Cultures

SRLVs isolation can be achieved through co-cultures of peripheral blood mononuclear cells (PBMCs) with sheep choroid plexus cells or goat synovial membrane cells [17]. The evidence of SRLVs infection is co-evaluated from the existence of a cytopathic effect and a positive reverse transcriptase activity assay [34]. However, the expected cytopathic effect (CPE), which is the formation of syncytia and/or refractile stellate cells with dendritic processes, may be difficult to detect for inexperienced staff with limited training in microscopy and cell biology. In addition, strain variability regarding the extend of detectable CPE cannot be excluded [67]. It is obvious that cell cultures cannot be routinely used for the diagnosis of SRLVs infections given the increased cost, the complexity, the limitations derived from in vitro viral replication, and the demands for specialized laboratory and trained personnel. Therefore, cell cultures are mainly applied either for the verification of the results of other molecular diagnostics or for research purposes in the fields of immunopathology and SRLVs genetics and molecular epidemiology [33,37,39,99,100]. 

## 3. Current and Future Perspectives in Diagnosis of SRLV Infections

Diagnosis of lentiviral infections constitutes the cornerstone for the successful implementation of eradication programs. A “gold standard” test with high values of sensitivity, specificity and accuracy, blindly used in every case does not seem readily feasible when considering the special characteristics of SRLVs (i.e., high genetic variability, mechanisms of virus replication, and animal humoral immune response). Nonetheless, the scientific community has addressed these limitations, proposing targeted combinations of diagnostic tools, which are constantly evaluated to reduce the possibility of both newly or persistently infected animals to evade diagnosis [27,39,62,63,70,82,86,101,102]. Although combination of diagnostics increases cost, time, and the effort required, it seems to be inevitable for the early and safe diagnosis in young animals which are likely infected but seronegative. However, in lambs early diagnosis may be limited by interference of maternal antibodies or provirus transmitted during suckling or milk aspiration [103].

Genotyping and classification of the circulating SRLVs strains in a specific region/breed could permit the targeted application of appropriate serological and molecular tests. In this direction, combined peptide ELISAs with type-specific epitopes from multiple genotypes could be tested in old and/or symptomatic animals and in mixed flocks (cross-species transmission and recombination) before the design of primers for PCR-based methods. Additionally, diagnostic tools should be adapted in a more animal- and farmer-friendly framework, utilizing biological materials with less invasive sample collection techniques. For example, milk is a promising alternative to blood and in many cases exhibits satisfying concordance with the results obtained from serum and whole blood samples both on serological and PCR assays; however, standardization of milk as sampling matrix and further verification in the field is needed for its use in serological and molecular tests. Particularly, in the case of highly sensitive screening tests, bulk milk samples could be incorporated as SRLVs-status determination tests for the initial characterization of a flock as SRLVs-infected or free. Newly developed technologies used in the HIV diagnosis such as specific antibody-antigen biomarkers or dried-blood spot testing [104] could be exploited in combination with LAMP and RPA-LFD techniques on SRLVs diagnosis for the development of in situ, rapid, user-friendly, cost-effective, and reliable diagnostic tools. In future, point-of-care (POC) testing of small ruminant infectious diseases in mobile platform technologies could integrate SRLVs diagnostic assays contributing to the control and elimination of critical epidemic and endemic diseases, including MV and CAE.

## 4. Impact of Early and Efficient SRLVs Diagnosis

Early and effective diagnosis of SRLVs and subsequently the control of MV and CAE are both critical endeavors for countries with a developed small ruminant farming sector. In addition, until now the applied programs for the eradication of SRLVs have not been scheduled based on a common diagnostic protocol, which allows deviations in the interpretation of requirements for the accreditation of SRLV-free regions and farms. Research on SRLVs diagnostics will form the steppingstone for the surveillance of the disease and the investigation of alternative control strategies. Linking the epidemiological characteristics of the disease with the use of novel and more efficient diagnostic techniques can ensure an integrated approach for the control of the disease in practice. 

The economic impact of SRLVs early diagnosis is likely enormous, as both MV and CAE are associated with dramatic, direct, and indirect economic losses, which undermine the sustainability of the farms. The magnitude of economic losses caused by the diseases is determined by factors related to their clinical symptoms and epidemiology at the farm level. The effective control of the diseases may drastically reduce monetary losses associated with the detrimental effects on health, welfare, and productivity of animals, while early diagnosis will facilitate for the first time the large-scale production of certified SRLVs-free breeding stocks, enjoying the expected added-value. The enhancement of the economic sustainability of farms will further facilitate the development of the sector and the eligibility of the small ruminant farming profession. It will also contribute to the survival of farmers in the provinces, particularly in disadvantaged and remote areas, where livestock farming is one of the most important, main, or complementary sources of income. Moreover, animal health and welfare status will be significantly improved via early diagnosis of SRLV infections, and the requirement for safe products of animal origin, produced by healthy animals that live “a life worth living”, will be satisfied.

## Figures and Tables

**Table 1:** Commercially available ELISA kits used for the diagnosis of SRLV infections.

Commercial Kit Product Name	ELISA Format	Antigen	Sample/ Diagnostic Matrix	Se/Sp	Reference Test	Ref
LSIVet^TM^ Ruminant Maedi-Visna/CAEV serum ELISA kit (LSI, Thermo Fisher Scientific, Waltham, MA, USA)	Competitive	gp135 TM protein/A and B genotypes	Serum	90.2% ^a^/92.8% ^a^ 100.0% ^b^/85.7% ^b^	qPCR	[27]
ID screen^®^ MVV/CAEV indirect (IDvet Innovative Diagnostics, Grabels, France)	Indirect	peptides from the MVV/CAEV, gp135 and p25 proteins/A, B and E genotypes	Serum, plasma and milk	100.0% ^a^/97.8% ^a^ 91.7–100.0% ^b^/97.6–98.9% ^b^	qPCR, ELISA ^A,B^	[27,64]
Eradikit™ SRLV screening test (IN3 diagnostic, Italia)	Indirect	*gag* and *env* peptides/A, B and E genotypes	Serum, plasma and milk	96.1% ^a^/99.4% ^a^ 100.0% ^b^/94.6% ^b^	qPCR	[27]
Elitest MVV/CAEV (Hyphen BioMed, Neuville-sur-Oise, France) or Innotest MVV (Innogenetics, Gent, Belgium)	Indirect	MVV capsid rp25 and gp46 TM protein/EV-1 strain, A genotype	Serum	98.0, 96.9, 97.8, 99.3% ^a^/94.7, 99.2, 98.2, 99.4% ^a^ 95.8% ^b^/99.7% ^b^	qPCR, Bayesian analysis, AGID and WB	[27,32,46,65]
MVV/CAEV p28 Ab Screening Test (Idexx, Westbrook, ME, USA)	Indirect	peptide of TM protein (*env* gene) and of the recombinant p28 capsid protein/A genotype	Serum and plasma	84.3% ^a^/99.6% ^a^ 91.7% ^b^/100.0% ^b^	qPCR	[27]
ELISA MAEDI VISNA/CAEV (Institut Pourquier, Montpellier, France) *	Indirect	recombinant p28 gag protein and peptide of the *env* protein (gp135)/A genotype	Serum	98% ^a^/97.4% ^a^	Bayesian analysis	[32]
CAEV/MVV Total Ab Test (Idexx, Westbrook, ME, USA) or Checkit CAEV/MVV (Dr. Bommeli AG, Bern, Switzerland)	Indirect	Whole virus/strain OLV, A gentoype	Serum, plasma and milk	98.6% ^b^/99.3% ^b^ 91.4% ^c^/98.9% ^c^	GAG-GST ELISA **	[66]
Small Ruminant Lentivirus Antibody Test Kit, cELISA (VMRD, Pullman, WA, USA)	Competitive	SU Antigen of gp135/B genotype	Serum	98.6% ^a^/96.9% ^a^ 100% ^b^/96.4% ^b^	RIPA	[43,44]
INgezim Maedi screening^TM^ (Ingenasa, Eurofins Technologies, Spain)	Indirect	synthetic peptides from the *env* protein/A and B genotypes	Serum	No published data		
Enferplex Goat/Sheep Multi-Disease 5D (Enfer Scientific, Co. Kildare, Ireland)	Indirect	recombinant p25 core protein, TM1 gp46 synthetic peptide	Serum, plasma and milk	No published data		

Se: sensitivity; Sp: specificity; ^a^ Sensitivity and specificity values for sheep; ^b^ Sensitivity and specificity values for goats; ^c^ Sensitivity and specificity values for milk samples; gp: glycoprotein; TM: transmembrane; Ref: reference. * before merge of Institut Pourquier by Idexx Laboratories in 2007; ** recombinant GAG (group-specific antigens)-GST (glutathione S-transferase) fusion protein expressed in *E. coli*; ^A^: Checkit CAEV/MVV monophasic Dr. Bommeli AG, Bern, Switzerland; ^B^: ELISA MAEDI VISNA/CAEV Institut Pourquier, Montpellier, France.

**Table 2:** PCR techniques and information regarding primers, detected strains, animal species, country and diagnostic performance.

PCR Type	Sample	Tarteted Genomic Region (Amplicon Size, bp)	Se (%)/Sp (%)	Concordance (%)/ *k* Value	Reference Method/Diagnostic Matrix	Animal Species	Country	Ref
c-PCR	DNA (PBLs, MCPs and TSs)	LTR (291 bp)	83.5%/100.0% (PBLs) 66.7%/100.0% (MCPs) 89.6%/100.0% (TSs) 97.7%/100.0% (overall)		AGID ^A^ and ELISA ^B^/BS	s	Spain	[70]
RT-PCR and c-PCR	DNA and RNA (PBLs, BS and BC)	*gag* gene (748 bp) for c-PCR and RT-PCR LTR (291 bp) for c-PCR	Complementary Se: -gag-PCR 0% (PBLs) and 24% (BC) -LTR-PCR 0% (PBLs and BC) -gag-PCR to LTR-PCR 54.6% (PBLs) -LTR-PCR to gag-PCR 0% (PBLs and BC)	gag-PCR: *k* = 0.68 (PBLs) *k* = 0.69 (BC) LTR-PCR: *K* = 0.52 (PBLs) *K* = 0.59 (BC) gag-PCR with LTR-PCR: *k* = 0.73 (PBLs) *k* = 0.50 (BC)	ELISA ^C^/BS	s	Spain	[61]
n-PCR	DNA (PBMCs)	*gag* gene (500 bp)	Se: 73.0% (s) and 86.0% (g)		ELISAs ^C,D^ and AGID ^A^/BS	s/g	Norway	[77]
n-PCR	DNA (WB)	*gag* gene (1191 bp and 1327 bp)	69.6%/100.0%		cell cultures/ isolated monocytes	g	Thailand	[78]
n-PCR	DNA (PBLs, PBMCs, andBM)	*env* gene (625 bp, 394 bp or 608 bp) *gag* gene (990 bp)		47.0%	ELISA ^D^/BS	s/g	Poland	[79]
Semi n-PCR	DNA (PBMCs)	*pol* gene (412 bp and 404 bp)	Complementary Se: 25%		AGID ^A^/BS	s/g	Greece	[80]
Semi n(RT)-PCR	RNA (blood and milk)	*pol* gene (475 bp and 303 bp)		62.0%/0.05 (ELISA and PCR in blood) 62.0%/0.2 (ELISA and PCR on milk)	ELISA ^C^/BP and milk	s/g	Spain	[48]
Semi n(RT)-PCR c-PCR	DNA and RNA (milk)	*pol* gene for semi n(RT)-PCR (475 bp and 303 bp) LTR for c-PCR(291 bp)	28.4%/68.9% (pol-PCR-s) 1.6%/95.5% (LTR-PCR-s) 53.9%/73.3% (pol-PCR-g) 7.7%/99.3% (LTR-PCR-g)	*k* = 0.02 (*pol*-PCR-s) *k* = 0.02 (LTR-PCR-s) *k* = 0.12 (*pol*-PCR-g) *k* = 0.11 (LTR-PCR-g) *pol*-PCR with LTR-PCR: 73.2%/0.02 (s) 73.4%/0.06 (g)	ELISA ^C^/milk	s/g	Spain	[81]
qPCR	DNA (BCCs)	*gag* gene (138 bp) LTR (291 bp)	Se: gag-PCR 88.0% (s) 82.0% (g) LTR-PCR 83.0% (s) 40.0% (g)	*k*: gag-PCR 0.73 (s)/0.37 (g) LTR-PCR 0.78 (s)/0.10 (g)	ELISA ^C^/BS	s/g	The Netherlands	[82]
qPCR	DNA (BCCs and MCPs)	*gag* gene (138 bp) LTR (291 bp)	LTR-PCR: 21.0%/- (BCCs-g) 50.0%/94.0%(MCPs-g) 82.0%/80.0% (BCCs-s) 70.0%/99.0% (MCPs-s) gag-PCR: 39.0%/- (BCCs-g) 61.0%/88.0% (MCPs-g) 89.0%/81.0% (BCCs-s) 89.0%/89.0% (MCPs-s)	between PCRs: 77.0%/0.45 (BCCs-g) 83.0%/0.63 (BCCs-s) 87.0% /0.76 (MCPs-g) 87.0%/0.75 (MCPs-s) 90.0%/0.76 (MCPS of bulk milk-g)	ELISA ^C^/BS	s/g	The Netherlands	[49]
qPCR	DNA (PBLs)	*gag*MA (113 bp) LTR (101 bp)	Se: 89.9% (*gag*MA-PCR) 80.2% (LTR-PCR) 79.0% (overall)	*k* = 0.72	ELISA ^E^/BS	s/g	Slovenia	[83]
qPCR	DNA(PBLs and BC)	*gag* gene (524 bp)	88.0%/100.0% (PBLs-s) 83.3%/100.0% (PBLs-g) 63.0%/100.0% (blood clot-s) 21.0%/100.0% (blood clot-g) 75.0%/100.0% (blood clot-s) 25.0%/100.0% (blood clot-g)		ELISA ^C,F,G,H,I^ and AGID ^J^/BS qPCR in PBLs	s/g	Belgium	[27]
qPCR	DNA (PBMCs and MCPs)	*gag* gene (524 bp)	relative values: 81.0%/88.0%	84.0% concordance	qPCR in PBMCs	g	Belgium	[47]
Semi n-qPCR	DNA (PBLs)	*pol* gene (455 bp and 416 bp)	Se: 86.0% Relative Se: 28.0%	79.0%/0.58	ELISA ^G^/BP	s/g	Greece	[84]
q(RT)-PCR	DNA (WB) and RNA (WB and BS)	*env* gene (114 bp)	qPCR: 83.3%/87.5% RT qPCR: 58.3%/66.6%		ELISA ^D,E^/BS	s/g	Italy	[85]
q(RT)-PCR	DNA and RNA (PBLs and lung samples)	*gag* gene (524 bp)	86.7%/80.0% 60.9%/75.0%	87.0% 61.0%	AGID ^A^/BS ELISA ^C^/BS	s/g	Βelgium	[86]

c-PCR: conventional PCR; n-PCR: nested PCR; qPCR: quantitative (real-time) time PCR; RT-PCR: reverse transcription PCR; PBLs: peripheral blood leukocytes; PBMCs: peripheral blood mononuclear cells; MCPs: milk cells pellets; TSs: tissue samples; BS: blood serum; BP: blood plasma; BC: blood clot; BCCs: buffy coat cells; WB: whole blood; BM: bronchoalveolar macrophages; bp: base pairs; Se: sensitivity; Sp: specificity; k value: kappa coefficient; s: sheep; g: goats; Ref: reference. ^A^: Maeditect, Veterinary Laboratory Agency, Weybridge, UK; ^B^: Innotest MVV, Innogenetics, Gent, Belgium; ^C^: ELITEST-MVV, HYPHEN BioMed, France; ^D^: VISNA-MAEDI/CAEV, Institut Pourquier, Montpellier, France; ^E^: Checkit CAEV/MVVELISA Bommeli AG/Idexx Laboratories, Liebefeld, Switzerland; ^F^: MVV/CAEV p28 Ab screening test, Idexx, Westbrook, ME, USA; ^G^: ID screen^®^ MVV/CAEV, IDVet Innovative Diagnostics, Grabels, France; ^H^: LSIVetTM Ruminant Maedi-Visna/CAEV serum ELISA kit LSI, Thermo Fisher Scientific, Waltham, MA, USA; ^I^: Eradikit™ SRLV, IN3 diagnostic, Italia; ^J^: AGID CAEV P28 kit, Idexx, Westbrook, ME, USA.

## Supplementary Materials

The following are available online at https://www.mdpi.com/article/10.3390/v13091711/s1, Table S1: Primer sequences of PCR techniques referred in Table 2.
