# Supplementary material for: Serological, Molecular and Culture-Based Diagnosis of Lentiviral Infections in Small Ruminants
Source: Viruses. 2021 Aug 27;13(9):1711. doi: 10.3390/v13091711 (PMC8473411; doi:10.3390/v13091711)
Supplement: Supplementary file 1 [file viruses-13-01711-s001.zip › viruses-1302299-supplementary.pdf]

Table S1. Primer sequences of PCR techniques referred in Table 2.

PCR type	Tarteted genomic region (amplicon size, bp)	Primers Sequences (5'-->3')	Ref
c-PCR, qPCR	LTR on GenBank accession n. M10608 (291 bp)	F: TGACACAGCAAATGTAACCGCAAG<br>R: CCACGTTGGGCGCCAGCTGCGAGA	[1–5]
Semi n-PCR	*Pol* gene from K1514, EV1, SA-OMVV, CAEV-Cork (1st round 412 bp & 2nd round 404 bp)	1st round<br>F: CARGGIGGIATMATAGAYTCIGG<br>R: ARTGIGTRTARTCIACYTGCCA<br><br>2nd round<br>F: GGGATMATAGAYTCGGGRTATCARGG<br>R: TGGGTRTARTCGACYTGCCARTG	[6]
qPCR	*Gag* gene on GenBank accession n. M51543 (138 bp)	F: TGGCGCCCAACGTGGGGC<br>R: CTTCAGGCGTCCCCGAAG	[2,3]
n-PCR	*gag* gene (500 bp) of CAEV-Co GenBank accession n. M33677	1st round<br>F: CTG GCG GCC CAA CGT GG<br>R: CAC AAG ACC ATG CTG CAT DGCYAC<br>2nd round<br>F: GAT AGA GAC ATG GCG AVG CAR G<br>R: AAGACC ATG CTG CAT DGC YAC TGT	[7]
RT-PCR	*Gag* gene (748 bp)	F: GGGACGCCTGAAGTAAGGTA<br>R: CAAAATCCTCGGACACAAG	[4]
q(RT)-PCR	*env* gene (114 bp) GenBank accession n. NC_001463	F: TACATGCGCTTAACTGGG<br>R: TACCTGRGGTYKATACTTAC	[8]

n-PCR	*env* gene (625 bp or 394 bp or 608 bp)<br>*gag* gene (990 bp) of CAEV-CO molecular clone	*env*-PCR<br>1st round<br>F: ACAAAGATGGCTWGCWATGCTTA<br>R: ATGCCAGCAATCCAATTCWTGGT<br>2nd round<br>C1V2<br>F: AGGTAAGTATAAACCCCAGGTAAG<br>R: GGCATCTTTTCTGTACAGGAGACTGCT<br>Or V1V2<br>F: TTGCAAAATGGGGATGTCAACC<br>R: GGCATCTTTTCTGTACAGGAGACTGCT<br>Or V4V5<br>F: GGIACIAAIACWAATTGGAC′<br>R: GCYAYATGCTGIACCATGGCATA<br><br>*gag*-PCR<br>1st round<br>F: TGGTGARKCTAGMTAGAGACATGG<br>R: GTTATTCCATAGGAGGAGCGGACGGCACCA<br>2nd round<br>F: GGAGCACTTGACAGAAGGAAA<br>R: GATCAGAAGGGTTCAAAATGAA	[9]
Semi n(RT)-PCR	*pol* gene from K1514 for semi-nested (475 bp & 303 bp)	1st round<br>F: DSAAGARAAATTARARGG<br>R: ATCATCCATRTATATBCCAAATTG<br>where B = C, G or T, D = A, G or T, R = A or G, and S = C or G<br>2nd round<br>replacing the F primer with<br>GATTTAACAGAGGCACA	[10]
Semi n(RT)-PCR	*pol* gene from K1514 for semi-nested PCR (475 bp & 303 bp)	pol-PCR<br>1st round<br>F: DSAAGARAAATTARARGG<br>R: ATCATCCATRTATATBCCAAATTG<br>2^{nd}round<br>replacing the F primer with<br>GATTTAACAGAGGCACA<br><br>-3′	[5]
qPCR	*gag*MA gene from CAEV Co (113 bp)<br><br>LTR from KV1514	*Gag*MA-PCR for g<br>F: GGGAAAAGGGATTATCCTGAG<br>R: GTTTTAAGGCACCAYAAACAATTTC<br>CAEV MA probe: TCTGTCAAGTKCTCCCCTCTG-<br>LTR-PCR for s<br>F: GGATACCCCGAGCTCAAAG<br>R: MVVMA R3 TTYAAKGCCCAYAGACARTT<br>MVV MA probe: TCTGTCAAGGTCTCCTTCCCG	[11]

	(101 bp)		
n-PCR	*gag* gene (1st round 1191 bp & 2nd round 1327 bp)	1st round<br>F: AACTGAAACTTCGGGGACGCCTG<br>R: GTTATCTCGTCCTAATACTTCTACTGG<br>F: AAGGTAAGTGACTCTGCTGTACGC<br>R: TTTTTCTCCTTCTACTATTCCYCC<br>2nd round<br>F: TGGTGAGTCTAGATAGAGACATGG<br>R: CAEV R2 GGACGGCACCACACGTAKCCC	[12]
qPCR	*gag* gene (524 bp) strain K1514	For genotype B:<br>F: AAWCCGCCRTGGTGARTCTAGATA<br>R: CSAGYTCAGGATAATCYCKTTTCC<br>SRLV-B probe: TGGCGAGGCARGTCTCCGG<br><br>For genotype A:<br>F: TAGAGACATGGCGAAGCAAGG<br>R: GCCCATAGACAGTTCCCTTC<br>MGB-probe: TACCCCGAGCTCAA	[13–15]
Semi n-qPCR	*pol* gene (1st round 455 bp & 2nd round 416 bp)	1st round<br>F: ZNA CARGGAGGAATMA-pdU AGAYICAGGATATCARGG-<br>R: ZNA CCYGAAT-pdU-WGTTTCTAYCCA<br>2nd round<br>F: CAGGGAGGAATMATAGAYGCAGGATAT<br>R: TCATAATGGGTRTARTCYACYTGCCAATG<br>Probe 1: AAA + T + G + GCATC + A + A + GATG<br>Probe 2: AAG + T + G + GCATC + A + A + GATG<br>Probe 3: AAA + T + G + GCATC + A + G + GATG	[16]

bp: base pairs; s: sheep; g: goat c-PCR: conventional PCR; n-PCR: nested PCR; qPCR: qunatitative (real time) PCR; RT-PCR: reverse transcription PCR; F:forward primer; R:reverse primer; IUPAC ambiguity codes: B = C, G or T; D = A, G or T; V = A, C or G; S = C or G; W = A or T; Y = C or T; R = A or G; M = A or C; K = G or T; I = inosine; ZNA5: Zipped nucleic acid primer conjugated with 5 cationic spermine units; pdU: 5-(1-propynyl)-2′-deoxy-uridine; Ref:Reference.

Reference

1. Extramiana, A.B.; González, L.; Cortabarría, N.; García, M.; Juste, R.A. Evaluation of a PCR technique for the detection of Maedi-Visna proviral DNA in blood, milk and tissue samples of naturally infected sheep. *Small Rumin. Res.* **2002**, *44*, 109–118, doi:10.1016/S0921-4488(02)00044-5.
2. Brinkhof, J.M.A.; van Maanen, C.; Wigger, R.; Peterson, K.; Houwers, D.J. Specific detection of small ruminant lentiviral nucleic acid sequences located in the proviral long terminal repeat and leader-gag regions using real-time polymerase chain reaction. *J. Virol. Methods* **2008**, *147*, 338–344, doi:10.1016/j.jviromet.2007.10.013.
3. Brinkhof, J.M.A.; Houwers, D.J.; Moll, L.; Dercksen, D.; van Maanen, C. Diagnostic performance of ELISA and PCR in identifying SRLV-infected sheep and goats using serum, plasma and milk samples and in early detection of infection in dairy flocks through bulk milk testing. *Vet. Microbiol.* **2010**, *142*, 193–198,

doi:10.1016/j.vetmic.2009.09.060.
4. Leginagoikoa, I.; Minguijón, E.; Berriatua, E.; Juste, R.A. Improvements in the detection of small ruminant lentivirus infection in the blood of sheep by PCR. *J. Virol. Methods* **2009**, *156*, 145–149, doi:10.1016/j.jviromet.2008.11.012.
5. Barquero, N.; Domenech, A.; Arjona, A.; Fernández-Garayzabal, J.F.; Ruiz-Santa-Quiteria, J.A.; Gomez-Lucia, E. Comparison of two PCR and one ELISA techniques for the detection of small ruminant lentiviruses (SRLVs) in milk of sheep and goats. *Res. Vet. Sci.* **2013**, *94*, 817–819, doi:10.1016/j.rvsc.2013.01.004.
6. Eltahir, Y.M.; Dovas, C.I.; Papanastassopoulou, M.; Koumbati, M.; Giadinis, N.; Verghese-Nikolakaki, S.; Koptopoulos, G. Development of a semi-nested PCR using degenerate primers for the generic detection of small ruminant lentivirus proviral DNA. *J. Virol. Methods* **2006**, *135*, 240–246, doi:10.1016/j.jviromet.2006.03.010.
7. Gjerset, B.; Rimstad, E.; Teige, J.; Soetaert, K.; Jonassen, C.M. Impact of natural sheep-goat transmission on detection and control of small ruminant lentivirus group C infections. *Vet. Microbiol.* **2009**, *135*, 231–238, doi:10.1016/j.vetmic.2008.09.069.
8. Brajon, G.; Mandas, D.; Liciardi, M.; Taccori, F.; Meloni, M.; Corrias, F.; Montaldo, C.; Coghe, F.; Casciari, C.; Giammarioli, M.; et al. Development and Field Testing of a Real-Time PCR Assay for Caprine. *Open Virol. J.* **2012**, *6*, 82–90.
9. Olech, M.; Rachid, A.; Croisé, B.; Kuźmak, J.; Valas, S. Genetic and antigenic characterization of small ruminant lentiviruses circulating in Poland. *Virus Res.* **2012**, *163*, 528–536, doi:10.1016/j.virusres.2011.11.019.
10. Barquero, N.; Arjona, A.; Domenech, A.; Toural, C.; De Las Heras, A.; Fernández-Garayzabal, J.F.; Ruiz-Santa Quiteria, J.A.; Gomez-Lucia, E. Paper: Diagnostic performance of PCR and ELISA on blood and milk samples and serological survey for small ruminant lentiviruses in central Spain. *Vet. Rec.* **2011**, *168*, 20, doi:10.1136/vr.c4951.
11. Kuhar, U.; Barlič-Maganja, D.; Grom, J. Development and validation of TaqMan probe based real time PCR assays for the specific detection of genotype A and B small ruminant lentivirus strains. *BMC Vet. Res.* **2013**, *9*, doi:10.1186/1746-6148-9-172.
12. Panneum, S.; Rukkwamsuk, T. Diagnosis of Caprine Arthritis Encephalitis Virus infection in dairy goats by ELISA, PCR and Viral Culture. *Pol. J. Vet. Sci.* **2017**, *20*, 347–353, doi:10.1515/pjvs-2017-0042.
13. Michiels, R.; Van Mael, E.; Quinet, C.; Adjadj, N.R.; Cay, A.B.; De Regge, N. Comparative analysis of different serological and molecular tests for the detection of small ruminant lentiviruses (Srlvs) in Belgian sheep and goats. *Viruses* **2018**, *10*, 8–10, doi:10.3390/v10120696.
14. Adjadj, N.R.; Vicca, J.; Michiels, R.; De Regge, N. (Non-)sense of milk testing in small ruminant lentivirus control programs in goats. Comparative analysis of antibody detection and molecular diagnosis in blood and milk. *Viruses* **2019**, *12*, doi:10.3390/v12010003.
15. De Regge, N.; Cay, B. Development, validation and evaluation of added diagnostic value of a q(RT)-PCR for the detection of genotype A strains of small ruminant lentiviruses. *J. Virol. Methods* **2013**, *194*, 250–257, doi:10.1016/j.jviromet.2013.09.001.
16. Chassalevris, T.; Chaintoutis, S.C.; Apostolidi, E.D.; Giadinis, N.D.; Vlemmas, I.; Brellou, G.D.; Dovas, C.I. A highly sensitive semi-nested real-time PCR utilizing oligospermine-conjugated degenerate primers for the detection of diverse strains of small ruminant lentiviruses. *Mol. Cell. Probes* **2020**, *51*, 101528, doi:10.1016/j.mcp.2020.101528.
